# The Acute Environment, Rather than T Cell Subset Pre-Commitment, Regulates Expression of the Human T Cell Cytokine Amphiregulin

**DOI:** 10.1371/journal.pone.0039072

**Published:** 2012-06-14

**Authors:** Yilin Qi, Darwin J. Operario, Steve N. Georas, Tim R. Mosmann

**Affiliations:** 1 David H. Smith Center for Vaccine Biology and Immunology, University of Rochester Medical Center, Rochester, New York, United States of America; 2 Department of Medicine, University of Rochester Medical Center, Rochester, New York, United States of America; New York University, United States of America

## Abstract

Cytokine expression patterns of T cells can be regulated by pre-commitment to stable effector phenotypes, further modification of moderately stable phenotypes, and quantitative changes in cytokine production in response to acute signals. We showed previously that the epidermal growth factor family member Amphiregulin is expressed by T cell receptor-activated mouse CD4 T cells, particularly Th2 cells, and helps eliminate helminth infection. Here we report a detailed analysis of the regulation of Amphiregulin expression by human T cell subsets. Signaling through the T cell receptor induced Amphiregulin expression by most or all T cell subsets in human peripheral blood, including naive and memory CD4 and CD8 T cells, Th1 and Th2 *in vitro* T cell lines, and subsets of memory CD4 T cells expressing several different chemokine receptors and cytokines. In these different T cell types, Amphiregulin synthesis was inhibited by an antagonist of protein kinase A, a downstream component of the cAMP signaling pathway, and enhanced by ligands that increased cAMP or directly activated protein kinase A. Prostaglandin E2 and adenosine, natural ligands that stimulate adenylyl cyclase activity, also enhanced Amphiregulin synthesis while reducing synthesis of most other cytokines. Thus, in contrast to mouse T cells, Amphiregulin synthesis by human T cells is regulated more by acute signals than pre-commitment of T cells to a particular cytokine pattern. This may be appropriate for a cytokine more involved in repair than attack functions during most inflammatory responses.

## Introduction

Different functional subsets of CD4 T cells are crucially involved in immune defense against diverse pathogens. At least four effector subsets are derived by differentiation from naïve CD4 T cells, and each expresses a characteristic combination of transcription factors, soluble mediators and surface molecules [1], [2]. Th1 cells predominantly produce interferon-γ (IFNγ) and protect against intracellular pathogens; Th2 cells produce interleukin (IL)-4, IL-5 and IL-13 and help to eliminate extracellular parasites; Th17 cells produce IL-17a and IL-17f and are crucial in fighting against extracellular bacteria and fungi; whereas induced T regulatory cells (iTreg) produce IL-10 and Transforming Growth Factor β (TGFβ), and suppress T and B cell effector responses.

Although the initial Th1 and Th2 subsets are relatively stable, recent studies have demonstrated some flexibility and plasticity, particularly in other CD4 T cell subsets. Cytokines and other soluble mediators in the lymph node and inflamed tissue can further affect the cytokine expression profile of effector CD4 T cells [3], in some cases by changing the differentiation status of the effector CD4 T cells. Primed precursor CD4 T (Thpp) cells, that produce mainly IL-2 and chemokines when stimulated, remain uncommitted with respect to their effector cytokine pattern and can later differentiate into either Th1 or Th2 cells [4]–[6]. Suppressive Treg cells, expressing the forkhead transcription factor Foxp3, can lose the expression of Foxp3 and acquire the ability to produce pro-inflammatory cytokines during autoimmunity [7]. Th17 cells can acquire the ability to produce IFNγ in Th1 polarizing conditions [8], [9]. Adoptively transferred IL-4-producing Th2 effector cells can produce IFNγ during viral challenge infections [10]. Th9 cells develop from Th2 populations in the presence of TGFβ [11], [12] and T follicular helper (Tfh) cells may represent a further differentiation step from several of the other subsets [13].

Acute modifications of cytokine patterns can also occur. IL-12+ IL-18 enhance the secretion of IFNγ by Th1 cells [14], [15], and IL-2 enhances cytokine production [16], [17]. In contrast, IL-10, TGFβ, prostaglandin E2 (PGE2) and adenosine inhibit inflammatory cytokine production [18]–[20].

Mouse Th2 cells, but not naive or Th1 cells, express Amphiregulin (AR), a member of Epidermal Growth Factor (EGF) family. Like other EGF members, AR is expressed as a transmembrane precursor protein and released by proteolytic cleavage [21], [22]. Soluble AR binds to EGF receptors and promotes proliferation and differentiation of epithelial cells, fibroblasts and keratinocytes [23]–[25]. AR-deficient mice [26] showed slower kinetics of clearance [27] of the helminth parasite, *Trichuris muris*, that is cleared most effectively by Th2-biased responses. AR production is also induced in human mast cells by IgE cross-linking [28], [29], in human eosinophils by IL-5 [30], and in human basophils by IL-3 [31]. Thus AR is induced by activation of at least four cell types contributing to Type 2 inflammation, suggesting a role for AR during an allergic immune response. In addition, production of AR by immune cells is potentially important for tissue remodeling and repair [21], [26] during and after damaging immune responses.

Very little is known about the regulation of AR gene expression in human T cells. We examined the regulation of AR synthesis by human T cells, and found that in contrast to mice, many subsets of human T cells, including CD4 and CD8, naive and memory, Th1 and Th2, all express AR in response to TCR stimulation. Factors that elevate cAMP levels synergized with TCR stimulation to enhance AR expression, while inhibiting expression of most inflammatory cytokines. Thus in human T cells, AR production is regulated strongly by the environmental context during stimulation, but not restricted to particular precommitted effector subsets of T cells.

## Materials and Methods

### Antibodies and Reagents

Biotinylated goat anti-human AR and biotinylated goat normal IgG (isotype control), and APC conjugated anti-human CCR4 (205410) were obtained from R&D Systems (Minneapolis, MN). LEAF™ purified anti-human CD3ε (OKT3), APC-Cy7 conjugated anti-human CD4 (RPA-T4), Pacific Blue or APC-Cy7 conjugated anti-human CD69 (FN50), PE-Cy5 conjugated anti-human CD154 (24–31), PerCP-Cy5.5 conjugated anti-human CD27 (O323), Alexa Fluor 700 conjugated anti-human CD62L (DREG-56), Pacific Blue conjugated anti-human CXCR3 (TG1/CXCR3), PE-Cy7 or PE-Cy5 conjugated anti-human CD123 (6H6), Alexa Fluor 700 conjugated anti-human IL-2 (MQ1-17H12), FITC conjugated anti-human IL-4 (MP4-25D2), and PerCP-Cy5.5 conjugated anti-human IL-17A (BL168) were purchased from BioLegend (San Diego, CA). Functional grade purified anti-human CD28 (CD28.2), PE conjugated anti-human CD45RA (HI100), FITC conjugated anti-human CD45RO (UCHL1), PE-Cy5 conjugated anti-human CD19 (HIB19), PE-Cy7 conjugated anti-human IFNγ (4S.B3), and APC-conjugated streptavidin were obtained from eBioscience (San Diego, CA). Alexa Fluor 488 conjugated anti-human CXCR5 (RF8B2) and PE-Cy7 conjugated anti-human CCR7 (3D12) were purchased from BD Bioscience (San Jose, CA). Qdot® 605 conjugated anti-human CD3 (UCHT1), PE-Texas Red and Qdot® 705 conjugated anti-human CD8α (3B5), PE-Texas Red conjugated anti-human CD4 (S3.5), TRI-COLOR and Qdot® 800 conjugated anti-human CD14 (TüK4), Qdot® 655 conjugated anti-human CD45RA (MEM-56), Pacific Blue conjugated anti-human TNFα (MP9-20A4), and LIVE?DEAD Fixable Yellow Dead Cell Stain Kit were obtained from Invitrogen (Carlsbad, CA).

7-Aminoactinomycin D (7-AAD) and TAPI-1 was obtained from Calbiochem (Gibbstown, NJ). cAMP agonist (8-CPT-cAMP) and cAMP antagonist (Rp-8-Br-cAMP) were purchased from BioLog (Bremen, Germany). Phorbol 12-myristate 13-acetate (PMA), ionomycin, monensin, PGE2, forskolin and 3-Isobutyl-1-methylxanthine (IBMX), adenosine were obtained from Sigma (St.Louis, MO).

### Human Peripheral Blood T cell Isolation and Activation

Heparinized blood was obtained from healthy donors under a protocol approved by the University of Rochester Medical Center Research Subjects Review Board. Written, informed consent was obtained from all subjects. PBMC were isolated by Ficoll-Hypaque (Cellgro, Herndon, VA) density gradient centrifugation. Cells were suspended in complete RPMI-8 (RPMI-1640 medium containing 100U penicillin/streptomycin (Invitrogen) supplemented with 8% heat-inactivated fetal calf serum (FCS, HyClone, Logan, UT)). In the experiment treating cells with adenosine, serum-free medium X-VIVO™ 20 (Lonza, Walkersville, MD) was used.

To purify human naïve and memory CD4 T cells from PBMC, fresh PBMC were stained with antibodies specific for cell surface markers and CD4+CD8-CD14-CD123-CD45RA+CD45RO- (naïve CD4 T cells) and CD4+CD8-CD14-CD123-CD45RA-CD45RO+ (memory CD4 T cells) were sorted on a FACSAria (BD Bioscience, San Jose, CA).

### *In vitro* Induction of Allogeneic Th1 and Th2 cell Lines

Purified human naïve CD4 T cells were stimulated with irradiated (100Gy) allogeneic Epstein-Barr virus (EBV) – transformed B cells (1∶1 ratio) in complete RPMI-8 medium at 10^5^ cells/mL in round-bottom 96-well plate. Th1-biased cultures contained recombinant human IL-2 (5 ng/mL, PeproTech), recombinant human IL-12 (20 ng/mL, PeproTech) and anti-IL-4 (5 µg/ml, R&D Systems). Th2-biased cultures contained recombinant human IL-2 (5 ng/mL), recombinant human IL-4 (20 ng/mL, R&D Systems), anti-IL-12 (5 µg/ml, ebioscience) and anti-IFNγ (5 µg/ml, R&D Systems). Fresh medium containing 5 ng/mL IL-2 was added if necessary to cultures showing strong proliferation. The cultures were restimulated and expanded every seven days.

To enrich for cells with the Th1 or Th2 phenotypes, after 14 days priming, Th1 and Th2 cells were stimulated with plate-bound anti-CD3+ anti-CD28 for 8 hours. IFNγ+ Th1 cells and IL-5+ Th2 cells were stained and sorted by the MACS cytokine secretion assay (Miltenyi Biotec, Auburn, CA) according to the manufacturer's instructions. The enriched IFNγ+ Th1 cells and IL-5+ Th2 cells were expanded as previously for 14 days.

### Intracellular Staining and Cell Surface Staining

For intracellular staining (ICS) of AR, PBMC (10^6^ per well) were stimulated with medium alone, anti-CD3 (5 µg/ml) + anti-CD28 (1 µg/ml), Staphylococcal enterotoxin B (SEB, 1 µg/ml), PMA (10 ng/mL) + ionomycin (500 ng/mL), influenza H1N1 peptides (H1N1 [New Caledonia/New York], 20 ng/mL/peptide), Fel d1 (50 µg/mL, INDOOR, Charlottesville, VA), Der p1 (50 µg/mL, INDOOR) or Tetanus peptides (3 µg/mL/peptide) in round-bottom 96-well plate (Costar, Corning Inc., Corning, NY). Th1 or Th2 cultures were treated with medium alone or PMA + ionomycin. After 10 hours stimulation (with 2 µM monensin present for the last 8 hours), the cells were first stained with LIVE?DEAD Fixable Yellow Dead Cell Stain Kit, and then stained for cell surface markers CD4, CD8, CD14, CD123, and CD45RA. After cells were fixed and permeabilized using Fix-Perm (BD Bioscience), the cells were stained with anti-AR, anti-IFNγ, anti-IL-2, anti-IL-4, anti-IL-17A, anti-TNFα, anti-CD3 and anti-CD69 (or anti-CD154) intracellularly.

For cell surface staining of AR, PBMC were stimulated with medium alone or 1 µg/ml SEB in the presence or absence of 50 µM TAPI-1, an ADAM17 protease inhibitor [32], for 6, 12, and 24 hours. After stimulation, the cells were stained with antibodies against AR, CD3, CD4, CD8, CD14, CD123, CD69 and 7-AAD. Data were acquired using an LSR II flow cytometer (BD Bioscience), and analyzed with FlowJo software (Tree Star Inc., Ashland, OR).

### Quantitative Real Time PCR for Gene Expression

Total RNA was extracted using TRIzol (Invitrogen) according to the manufacturer’s instructions. cDNA was prepared by reverse transcription from total RNA using MultiScribe™ Reverse Transcriptase (Applied Biosystems, Foster City, CA) with random hexamer primers (Applied Biosystems). Quantitative real-time PCR (RT-PCR) was performed using the Applied Biosystems 7900HT Sequence Detection System. Primers and probes specific for AR, Heparin-binding EGF-like Growth Factor (HB-EGF), IL-2, IFNγ, IL-3, IL-4, IL-5, IL-10, IL-13, CD3d, EGF, Neuregulin (NRG) 1–4, epiregulin (EREG), betacellulin (BTC) and TGFα were all obtained from TaqMan Gene Expression Assays (Applied Biosystems). CD3d gene expression was used as an endogenous control for normalizing mRNA amounts. All samples were run in duplicate and data were analyzed using SDS software (Applied Biosystems).

### Measurement of AR Release

Purified CD4 T cells were treated with medium alone or CD3/CD28 beads (cells:beads 2∶1) in the presence or absence of 50 µM TAPI-1. After 24 hours, the supernatants were collected and AR was measured using the human Amphiregulin DuoSet ELISA Development kit (R&D Systems). The detection limit of the assay was 7.8 pg/mL. Because the antiserum for the ELISA was produced by immunization with bacterial recombinant human AR, and the standard is also non-glycosylated AR, this ELISA probably underestimates the concentration of normal human glycosylated AR.

## Results

### 1. T cell Activation Induces Rapid AR Expression by Human T Cells

Mouse Th2 cells produce AR in response to TCR-mediated activation [27], and the expression of AR by hemopoietic cells contributes to the clearance of a helminth parasite. However, our recent studies showed that basophils were the major human PBMC type that produced AR in response to anti-CD3/CD28 stimulation [31], whereas production of AR by T cells was much lower. Therefore we examined human T cells in more detail, to determine whether human T cells could produce AR, and if so, whether this was produced preferentially by human Th2 cells.

**Figure 1:**
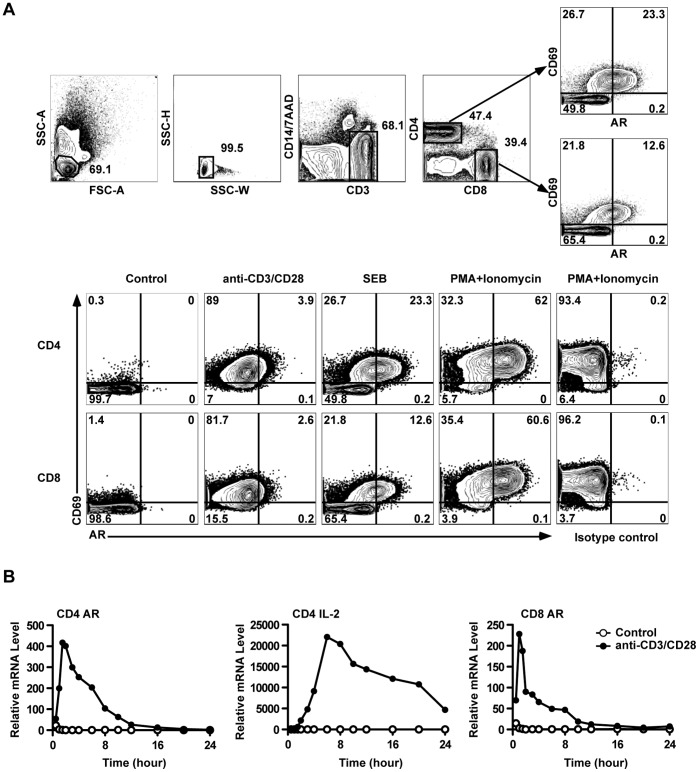
TCR activation induced AR expression in human PBMC T cells. (A) PBMC were treated as indicated and analyzed by ICS. The upper panels show the gating strategy to identify activated (CD69+) CD4 or CD8 T cells expressing AR. The lower panels show the induction of AR by different stimuli in CD4 or CD8 T cells. (B) AR and IL-2 mRNA were measured by RT-PCR in purified CD4 and CD8 T cells after activation by anti-CD3+anti-CD28 beads. Results in (A) and (B) are representative of at least three experiments.

Human PBMCs were stimulated with soluble anti-CD3+ anti-CD28, SEB, or PMA + ionomycin for 10 hours (protein secretion inhibitors were added during the last 8 hours). CD69 staining increased on almost all anti-CD3/CD28- and P+I-stimulated cells, and a subset of SEB-stimulated cells (Figure 1A). AR staining was increased, only in the CD69+ population, and this increase was most obvious in the P+I-stimulated cells. For all three stimulation conditions, the staining intensity for AR increased for the whole CD69+ population, i.e. separate positive and negative populations were not resolved, and so the percentage of cells in the AR+ gate may be an underestimate of the total number of cells expressing AR. The specificity of AR staining was demonstrated by using a control goat antiserum (right column). Similar results were obtained with CD8 T cells (Figure 1A).

To independently confirm AR expression by human T cells, and to test whether T cells produced AR as a direct result of TCR stimulation, human CD4 and CD8 T cells were purified by sorting, and stimulated with beads coated with anti-CD3+ anti-CD28 antibodies. At different times, RNA was extracted from the cells, and levels of AR and IL-2 mRNA measured by RT-PCR. AR mRNA levels increased rapidly after stimulation, and returned to low levels after ten hours, whereas IL-2 showed slower kinetics (Figure 1B). The kinetics of AR production were similar in CD4 and CD8 T cells. Thus human T cells directly express AR in response to polyclonal TCR stimulation.

### 2. Expression of Other EGF Family Members by Human T Cells

**Figure 2:**
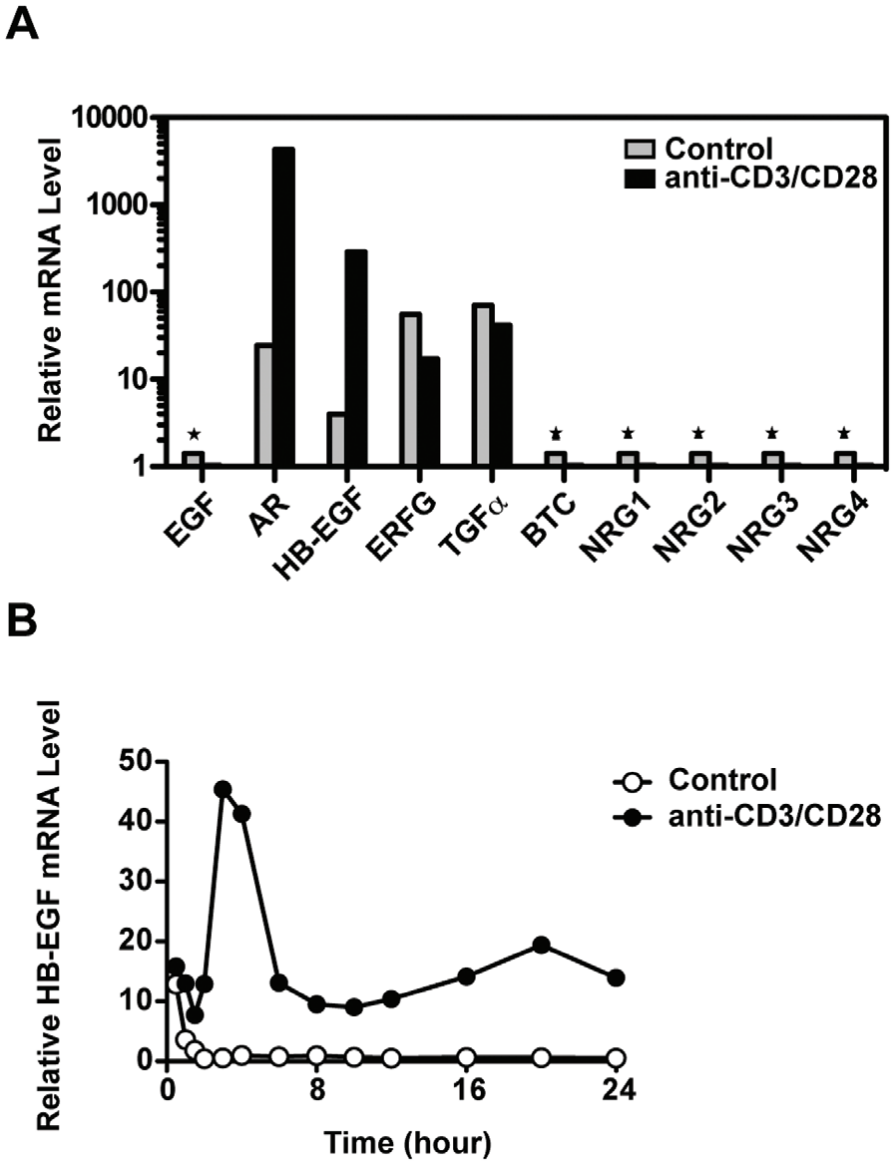
HB-EGF was also expressed by TCR activated human CD4 T cells. (A) Purified CD4 T cells were incubated with medium alone or anti-CD3/CD28 beads. At 4 hours, the mRNA levels of EGF family members were measured by RT-PCR. * Indicates undetectable values. (B) The kinetics of HB-EGF mRNA expression were measured on purified CD4 T cells responding to anti-CD3/CD28 beads. All results are representative of three independent experiments.

As demonstrated by other studies [33], HB-EGF mRNA was also upregulated in activated human CD4 T cells (Figure 2A), although the levels were lower than AR and peaked at a later time (Figure 2B). TGFα and EREG mRNA were also detected in resting CD4 T cells, but not increased during TCR activation. Other EGF members were undetectable. In our previous mouse experiments [27], AR and HB-EGF were also the only EGF family members induced by TCR stimulation (data not shown). Expression of HB-EGF protein was confirmed by cell surface and intracellular staining (data not shown).

### 3. Kinetics of AR Surface Expression and Release

**Figure 3:**
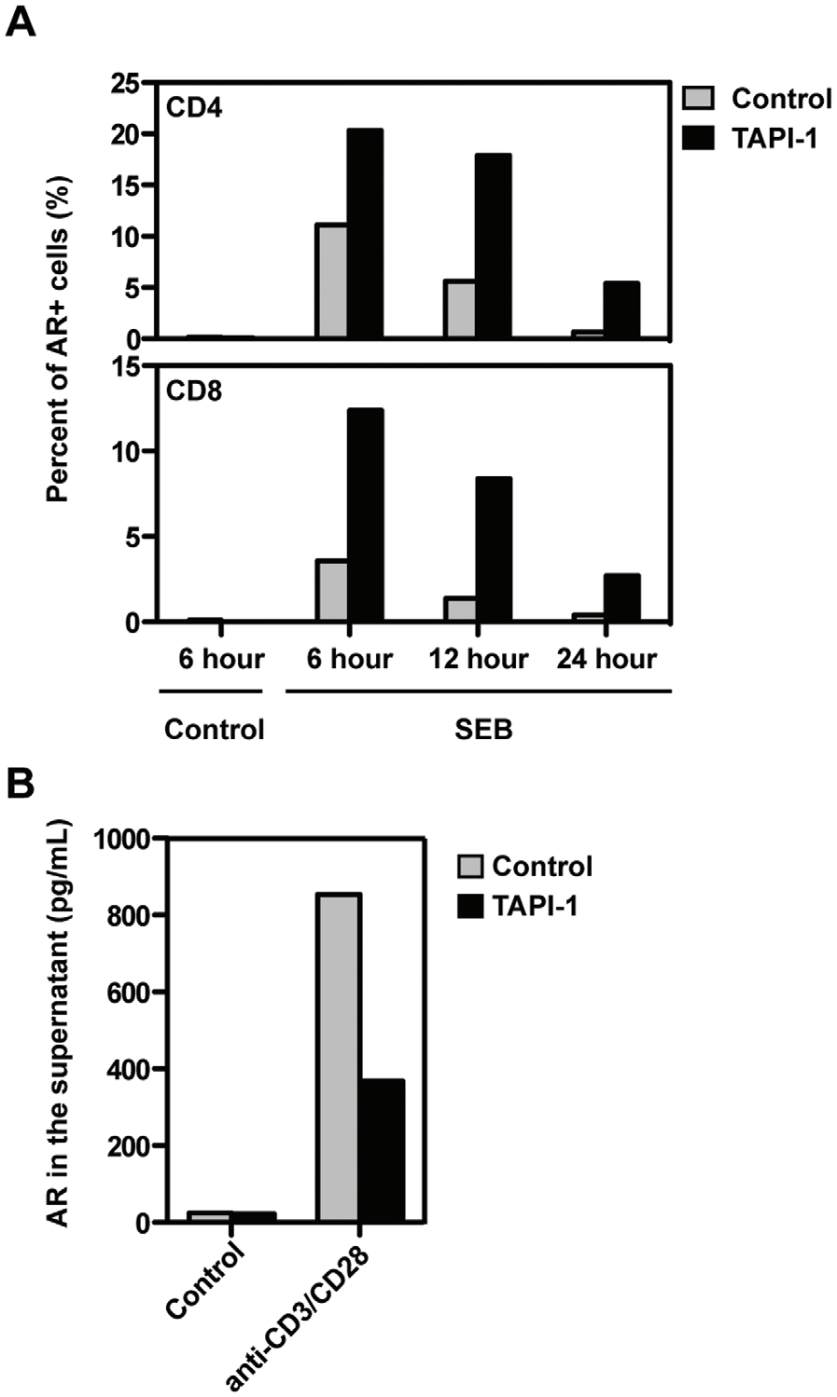
Release of AR from the T cell surface was blocked by the ADAM17 inhibitor TAPI-1. (A) PBMC were stimulated with SEB in the presence or absence of TAPI-1 for variable times. After cell surface staining of AR, the percentage of CD69+AR+ cells within CD4 and CD8 T cells was analyzed. (B) Purified CD4 T cells were treated with medium alone or anti-CD3/CD28 beads with or without TAPI-1 for 24 hours. The concentration of AR in the supernatant was measured by ELISA. All results are representative of at least three experiments.

EGF family members (including AR) are initially expressed as transmembrane proteins and released into the extracellular region after cleavage by metalloproteases, particularly ADAM17 [22]. To determine whether T cells also initially expressed surface AR and then released the soluble cleavage product, surface AR was stained during TCR activation in the presence or absence of the ADAM17/TACE inhibitor TAPI-1 [32]. TAPI-1 increased AR expression on the surface of both CD4 and CD8 T cells measured by frequency (Figure 3A) or fluorescence intensity (data not shown). Conversely, TAPI-1 decreased soluble AR in the supernatant (Figure 3B). In the absence of TAPI-1, AR expression on T cells gradually decreased and was barely detectable after 24 hours. As ADAM17 mRNA was detected by RT-PCR in resting human T cells and upregulated on activation (data not shown), these results suggested that AR was first synthesized as a membrane protein on human T cells and then released by ADAM17 cleavage, as in other cell types [34], [35].

### 4. Most or all T Cell Subsets can Express AR

In mice, AR was expressed selectively in TCR-activated Th2 cells [27] but not Th1 (Figure S1) or naive CD4 T cells. This was a pre-committed, intrinsic property of the Th2 cells, as Th2 but not Th1 cells expressed AR even when *in vitro*-derived mouse Th1 and Th2 cell lines were activated together in the same culture (data not shown). However, most human CD4 (and CD8) T cells expressed AR in response to PMA plus ionomycin stimulation (Figure 1A). We therefore examined in more detail which human T cell subsets were responsible for AR production.

#### Naive and memory CD4 and CD8 T cells produce AR

**Figure 4:**
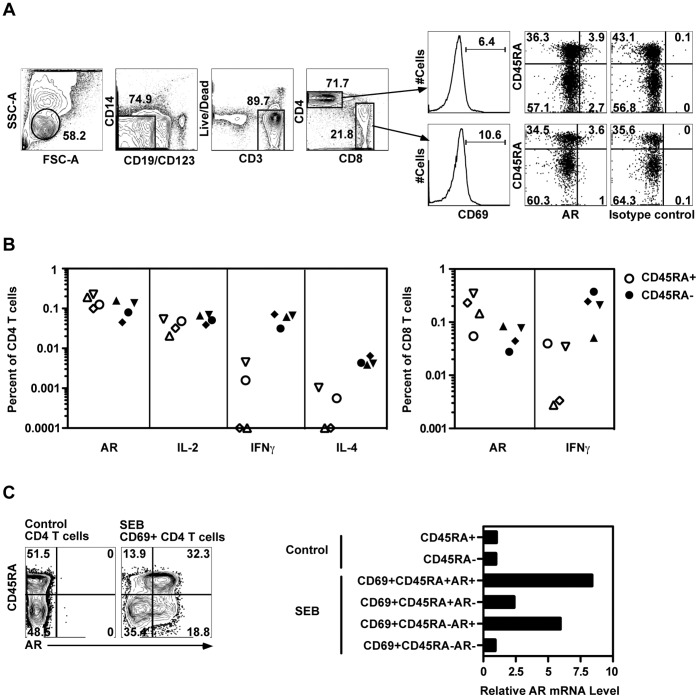
Both naïve and memory human CD4 T cells expressed AR during TCR activation. (A) PBMC were treated with medium alone or allogeneic EBV-transformed B cells for 10 hours and analyzed by ICS. The gating strategy to identify activated CD4+ and CD8+ T cells is shown. (B) AR, IL-2, IFNγ or IL-4 expression was measured in four subjects in CD45RA+ (open) and CD45RA- (solid) CD4+ and CD8+ T cells after allogeneic EBV-transformed B cell stimulation. Background values have been subtracted. (C) PBMC were treated with medium alone or SEB in the presence of TAPI-1 for 8 hours. Then six populations were sorted based on surface AR, CD69 and CD45RA expression (left). AR mRNA in each population was measured by RT-PCR (right). Results in (A) and (B) represent at least three experiments, (C) represents two experiments.

To examine the ability of naive and memory T cell subpopulations to express AR, we stimulated PBMC with an allogeneic EBV-transformed B cell line, which would be expected to activate a small fraction of both memory and naive CD4 and CD8 T cells. Alloantigens stimulated a fraction of both CD4 and CD8 T cells to produce AR (Figure 4A), relative to the unstimulated control. The specificity of staining was confirmed by isotype control antibodies. The cells producing AR (and other cytokines) were included in the CD69+ population.

AR was induced by allogeneic stimulation in both CD45RA- and CD45RA+ subsets of CD4 and CD8 T cells at frequencies ranging from 0.028% to 0.35%. These levels were comparable to the frequencies of CD4+ CD45RA+ or CD45RA- T cells producing IL-2, or CD4+ CD45RA- memory T cells producing IFNγ. As expected, IL-2 was produced by both memory (CD45RA-) and naive (CD45RA+) CD4 T cells, whereas IFNγ (and IL-4 at low levels) were produced mainly by memory cells (Figure 4B).

The expression of AR by both CD45RA- and CD45RA+ subsets of CD4 T cells was tested at the mRNA level in SEB-stimulated cells sorted according to AR and CD45RA expression (Figure 4C). Confirming the specificity of the anti-AR antibody staining, AR mRNA was enriched in AR+ cells from either CD45RA- or CD45RA+ populations.

#### AR is produced by memory CD4 T cell subsets expressing different cytokine phenotypes

**Figure 5:**
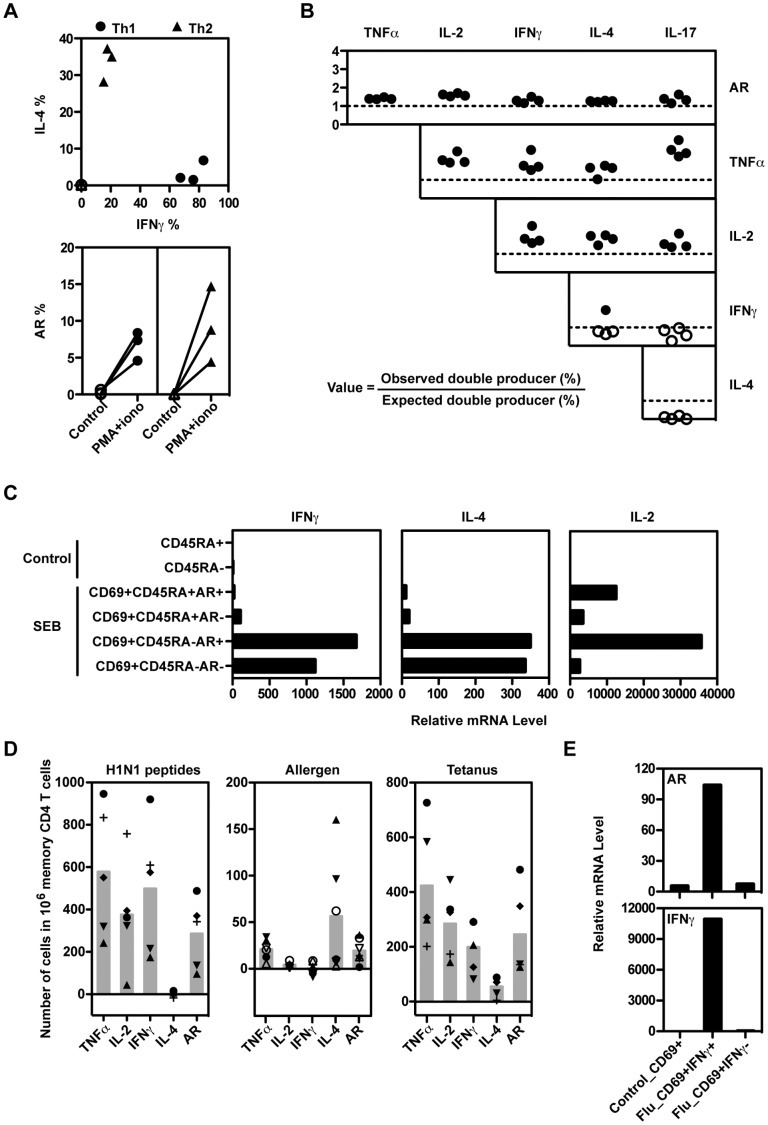
Several human CD4 T cell subsets can produce AR. (A) Allogeneic Th1 and Th2 cell lines from three subjects were stimulated with PMA + ionomycin for 6 hours. The percentage of cells expressing IFNγ, IL-4, and AR was analyzed by ICS. (B) The expression of AR and other cytokines was measured in SEB-stimulated PBMC from four subjects by ICS, calculating the frequencies of single cytokine producers, and all possible combinations of double-producers, among the CD154+ CD4+ T cells. The figure shows the ratio between the observed frequencies of double-producing T cells for each cytokine pair, and the expected frequencies (calculated as the product of the individual frequencies for each cytokine). Values represent the ratios for the double-producer combination defined by the row and column labels. Ratios above or below 1 are indicated by solid or open symbols, respectively. (C) IL-4, IFNγ and IL-2 mRNA levels were measured by RT-PCR in the sorted populations described in Figure 4C. (D) PBMC were treated with influenza H1N1 peptides or tetanus (five subjects each), or the allergens Fel d1 (solid symbols) or Der p1 (open symbols)(three subjects each). The numbers of memory CD4 T cells expressing AR and other cytokines were measured by ICS. The backgrounds (no antigen) have been subtracted. Each symbol represents one individual and the filled bar is the mean of all tested subjects. (E) CD69+ CD4+ T cells (Control_CD69+) were sorted from PBMC incubated in medium alone. CD69+IFNγ+ and CD69+IFNγ- CD4 T cells were sorted from influenza peptide-treated PBMC using the cytokine secretion assay. The mRNA levels of IFNγ and AR were measured by RT-PCR. Results in (A-C) are representative of at least three experiments, (D) represents two experiments using a total of 5 independent subjects, and (E) represents two experiments.

Although naive CD4 T cells are relatively homogeneous, the memory population includes a wide range of differentiated effector subsets. As AR is expressed selectively by mouse Th2 cells, we examined whether AR production by human CD4 memory T cells was preferentially associated with expression of a particular cytokine or surface marker pattern. Th1- and Th2-biased human CD4 T cell populations were induced by stimulation of sorted naive human CD4 T cells with an allogeneic B cell line in Th1- or Th2-biasing cytokine conditions. The populations were further enriched by using the Cytokine Secretion Assay to sort IFNγ- or IL-5-producing cells, respectively. The resulting populations were strongly polarized, but unlike mouse T cells, both Th1 and Th2 human cell lines expressed AR (Figure 5A).

These results were confirmed using *ex vivo* human CD4 T cell populations. Human PBMC were stimulated with SEB, and AR and other cytokines measured by intracellular staining. Naive cells (CD45RA+) expressed high levels of IL-2 and AR, but very low levels of either IFNγ or IL-4 (data not shown). Memory cells produced all cytokines tested, at varying frequencies. To determine whether AR expression was associated positively or negatively with subset-specific cytokines, the frequencies of cells expressing AR plus each of the other cytokines were measured from the ICS results. These values were then compared with the double-producing frequencies predicted for random association of each cytokine pair, by multiplying the individual frequencies for each cytokine. Figure 5B shows that AR was expressed in association with TNFα, IL-2, IFNγ, IL-4 and IL-17 at slightly higher frequencies than predicted by random association. Similarly, TNFα and IL-2 showed positive associations with all other cytokines. In contrast, the subset-specific cytokines IFNγ, IL-4 and IL-17 showed mostly negative associations between each other, as expected. These results were confirmed at the RNA level by sorting SEB-stimulated human PBMC according to surface AR expression. Both AR+ and AR- memory CD4 T cell populations expressed similar levels of IL-4 and IFNγ as measured by RT-PCR (Figure 5C). IL-2 mRNA levels were higher in AR+ T cells, in both CD45RA- and CD45RA+ cells.

#### AR is produced in response to antigen stimulation

We next tested whether human CD4 T cells expressed AR during antigen/APC stimulation in response to influenza peptides, allergens or tetanus antigens to stimulate Type 1, Type 2 and Thpp-biased recall responses, respectively [6]. PBMCs were stimulated with antigens for 10 hours, and AR and other cytokines measured by ICS.

Although these three antigens induced *in vivo* recall responses with characteristically different levels of IL-2, IFNγ and IL-4, all three antigens induced substantial production of AR in the activated (CD154+) cells (Figure 5D). Similar results were obtained with cells from multiple subjects, although the magnitudes of the antigen responses were variable for all cytokines. Thus AR can be expressed by all the conventional defined subsets of T cells that we have tested, including CD4 and CD8, naïve and memory, Thpp, Th1 and Th2.

To confirm the protein results, influenza-specific CD69+ IFNγ+ cells were sorted from two subjects (results from one subject are shown in Figure 5E), and RT-PCR demonstrated that AR mRNA levels were strongly elevated in the IFNγ+ influenza-specific cells compared to either CD69+ CD4+ cells from unstimulated cultures, or CD69+ IFNγ- cells from stimulated cultures. The specificity of the sorting was demonstrated by the strong enrichment of IFNγ mRNA in the CD69+ IFNγ+ population (Figure 5E).

#### AR is produced by T cell subsets expressing different chemokine receptors and surface markers

**Figure 6:**
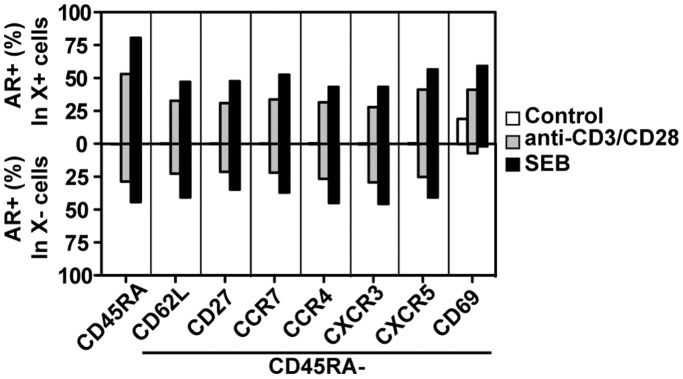
AR is produced by T cell subsets expressing different chemokine receptors and surface markers. PBMC were treated with medium alone, anti-CD3+ anti-CD28 antibodies, or SEB in the presence of TAPI-1 for 8 hours. Cells were stained for AR and cell-surface markers and analyzed by flow cytometry. Representative of two experiments.

Chemokine receptors expressed selectively by T cell subsets lead to different homing and chemotactic properties. Expression patterns of chemokine receptors are partly but not entirely related to cytokine commitment patterns [36]–[39]. Additional surface markers, including CD27 and the homing receptor CD62L are also expressed heterogeneously on human CD4 T cells. We therefore examined AR expression within subsets of memory CD4 T cells defined by the expression of these proteins. AR was produced at approximately similar frequencies by CD4 T cells positive or negative for the chemokine receptors CCR4, CCR7, CXCR3 and CXCR5, as well as CD62L and CD27 (Figure 6). However, expression of the activation-induced protein CD69 was strongly correlated with AR expression, as seen in previous figures. Taken together with the data described above, AR expression appears to be a general ability of most or all subtypes of human T cells after TCR activation.

### 5. Signaling through the cAMP/PKA Pathway Synergizes with TCR Signals to Induce AR

As our results had demonstrated that AR production was not limited to a pre-committed subset of T cells, we then tested whether AR production was regulated by acute signals in the immediate milieu during TCR stimulation. In many cell types AR is strongly regulated by the cAMP-PKA-CREB signaling pathway. AR expression was significantly up-regulated by cAMP-elevating agents in both resting and anti-CD3 stimulated human PBMC populations enriched for human T cells [40]. However, in that study the negatively-selected T cell population would also have contained basophils, and we have shown that basophils express AR rapidly in response to IL-3 and cAMP agonist ([31] and unpublished data). Thus anti-CD3 stimulation of the CD4 T cell + basophil population could have induced IL-3 production by T cells, indirectly resulting in AR production by basophils. We have now re-examined the effect of cAMP elevation on the expression of AR by different T cell subsets.

#### TCR and cAMP signals synergize to induce AR expression

**Figure 7:**
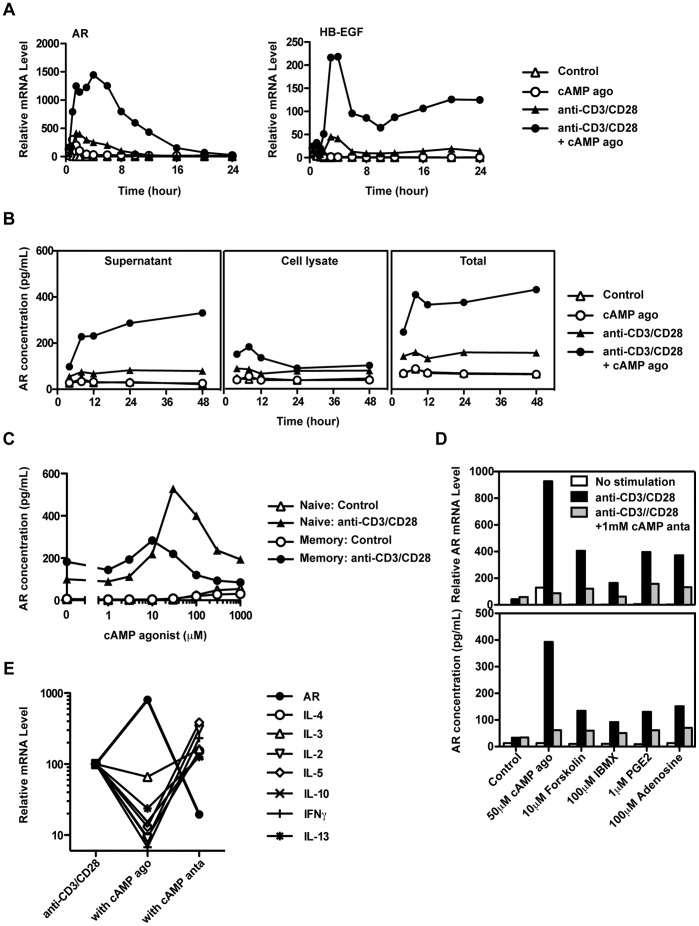
TCR and cAMP synergize to induce AR production in human CD4 T cells. Purified CD4 T cells were incubated with or without TCR stimulation (anti-CD3/CD28 beads) and the cAMP agonist. (A) AR and HB-EGF mRNA expression was measured by RT-PCR. (B) The concentrations of AR in the supernatant and cell lysates were measured by ELISA. (C) Enriched CD45RA+CD45RO- (naïve) and CD45RA-CD45RO+ (memory) CD4 T cells were treated with medium alone, or anti-CD3/CD28 beads in the presence or absence of cAMP agonist (1 ∼ 1000 µM). The concentration of AR in the supernatant at 24 hours was measured by ELISA. (D) Purified CD4 T cells were treated with medium alone, or anti-CD3/CD28 beads in the presence or absence of the cAMP-modifying agents shown. RNA was extracted at 4 hours, and AR mRNA was measured by RT-PCR. The concentration of AR in the 24-hour supernatant was measured by ELISA. (E) PBMC were treated with anti-CD3+ anti-CD28 antibodies in the presence or absence of cAMP agonist or antagonist for 8 hours. CD4 T cells were purified by cell sorting and RNA was extracted. The mRNA levels of AR and other cytokines were measured by RT-PCR. All results are representative of at least three experiments.

TCR activation alone (which transiently elevates cAMP [41]) induced transient AR mRNA expression (Figure 7A), and a strong cAMP agonist (PKA activator 8-CPT-cAMP) also induced low levels of AR in the absence of other signals. However, TCR and PKA signaling synergized to induce higher and more sustained levels of AR and HB-EGF mRNA (Figure 7A), as well as high levels of AR protein (supernatant plus cell-associated, Figure 7B). This strong synergy contrasts with a previous study [40], possibly due to the presence of basophils in the responding population in that study.

As both naive and memory CD4 T cells produce AR (Figure 4), we tested whether PKA activation would enhance AR expression in both populations. Figure 7C shows the response of purified CD45RA+ and CD45RA- CD4 T cells to anti-CD3/CD28 stimulation. The cAMP agonist strongly enhanced AR expression in both populations, with a slightly higher optimal concentration in naive CD4 T cells (30 µM) compared to memory CD4 T cells (10µM).

#### AR expression is modified by natural and synthetic modulators of cAMP signaling

In the experiments described above, cAMP signaling was altered by an agonist (8-CPT-cAMP) that directly targeted PKA to mimic the increase of intracellular cAMP levels. To further confirm that the cAMP-PKA-CREB signaling pathway regulates AR expression, we tested natural and pharmacological agents that increase the intracellular levels of cAMP by acting at two additional steps: PGE2 and adenosine are natural ligands for G-protein coupled receptors that activate adenylyl cyclase [20], [42]–[44]; forskolin activates adenylyl cyclase directly [20]; and IBMX is a broad inhibitor of cAMP-degrading phosphodiesterases [45].

Consistently, all four cAMP elevating agents upregulated AR mRNA and protein expression in anti-CD3-stimulated T cells. In each case, the elevated signal was blocked by the cAMP antagonist (Figure 7D). The enhancement of AR by the PDE inhibitor suggested that PDE reduced the moderate levels of cAMP induced by TCR activation in CD4 T cells [41].

#### AR and other cytokines are regulated reciprocally by cAMP signals

In contrast to the enhancement of AR expression, the cAMP agonist inhibited expression of many other cytokines (Figure 7E), and all four PKA-activating agents described in Figure 7 inhibited expression of IL-2 and IFNγ (data not shown). These results are consistent with previous studies with cAMP agonists and natural cAMP elevating agents, such as PGE2 and adenosine [19], [20]. Thus AR expression in T cells is enhanced under conditions that suppress the production of many other cytokines.

## Discussion

In contrast to the preferential expression of AR by mouse Th2 cells, we have now shown that synthesis of human AR is not restricted to a particular human T cell subset. AR can be produced by activated naive and memory CD4 and CD8 T cells, including Th1 and Th2 phenotypes. Our results suggest that AR is not a specific product of certain pre-committed effector subsets of human CD4 T cells, but instead is regulated mainly by additional signals present during T cell activation, particularly signals influencing the cAMP signaling pathway. The lack of precommitment suggests that, in contrast to the memory of effector functions carried by T cells committed to Th1, Th2, Th17 etc phenotypes, the amount of AR produced in a particular immune response is regulated by the local environment during that response, but is less influenced by previous immune priming. The discrepancy we have identified between mouse and human T cell regulation highlights the importance of performing cross-species comparisons of effector T cell phenotypes.

AR production was not restricted to a defined T cell effector subset, but AR and IL-2 levels were moderately correlated in both naïve and memory CD4 T cells. Although this could indicate the existence of a previously-unrecognized subset, it is possible that the correlation could be the result of shared transcriptional or mRNA stability regulatory factors, or to similar activation thresholds for IL-2 and AR. Expression of AR also showed moderate correlation with the expression of TNFα.

High levels of AR mRNA and protein were induced by synergy between TCR signals and signals that elevated cAMP or activated PKA. This contrasts with a previous report suggesting that both resting and anti-CD3 stimulated T cells significantly up-regulated AR in response to a cAMP agonist [40]. However, the enriched T cell population used in that study was purified by negative selection and very likely included basophils, which we have shown are potent producers of AR in response to IL-3 [31]. AR expression is also strongly enhanced by cAMP agonists in basophils (Y. Qi and T.R. Mosmann, unpublished data) and so it is possible that basophils may have produced the AR in response to the cAMP agonist without TCR stimulation.

In contrast to the induction of AR by cAMP elevating agents, these mediators suppress inflammatory responses by inhibiting cytokine expression and T cell proliferation. Synthesis of several pro-inflammatory or Type 1 cytokines is inhibited by cAMP (Figure 7E and [19], [20], [46], [47], whereas cAMP can either inhibit or enhance production of Type 2 cytokines such as IL-4, IL-5 and IL-13 (Figure 7E and [47]) depending on the stimulation conditions [48], [49].

Natural mediators that elevate the cAMP pathway and lead to PKA activation include PGE2 (mainly via the G protein-coupled receptors E2 and E4 on T cells) and adenosine (mainly via the A_2A_ receptor on T cells). Both mediators are produced at sites of immune inflammation, adenosine by degradation of ATP from dying cells, and PGE2 by activated macrophages. PKA activation signals also synergized with TCR signals to induce HB-EGF mRNA and protein expression in human CD4 T cells (data not shown). Thus during the progression of an inflammatory response, there may be a switch from pro-inflammatory cytokine production to AR (and HB–EGF) production.

Our findings allow us to construct a model of the role of T cell derived AR in adaptive immunity. During an immune response, initial immune attack mechanisms that destroy the pathogen are superseded at later times by suppression that reduces immunopathology, and tissue repair that restores normal structure and function. T lymphocytes are major cellular contributors to all three phases, and are thought to play a role in repair by producing HB–EGF and bFGF [33]. AR and HB–EGF, as members of the EGF family, promote the proliferation of fibroblasts, epithelial cells, and smooth muscle cells, which are major cell types repaired or remodeled at local tissue sites during an inflammatory response. Tissues with chronic inflammation show extensive cell proliferation, tissue thickening and reduced elasticity. Regulation of the balance between attack and repair cytokines produced by T cells is thus crucial to the successful outcome of the response.

In this model, AR derived from human T cells would be expressed mainly in response to tissue injury, consistent with the importance of the local environmental signals for AR regulation. This contrasts with the requirement for specific effector mechanisms to combat different pathogens, in which pre-commitment to cytokine effector phenotypes (thus linking antigen and effector specificities) may be more effective for regulating clearance functions. Collectively, the coordinate inhibition of pro-inflammatory cytokines and induction of tissue-remodeling cytokines of the EGF family may represent a switch from pathogen clearance to tissue repair mechanisms by effector human T cells.

## Supporting Information

Figure S1 **Mouse Th2 but not Th1 cells express AR in response to TCR activation.** *In vitro* induced allogeneic Th1 and Th2 cell lines [50] from B6PL or AR^−/−^ mice were stimulated with plate-coated anti-CD3 (2 µg/mL) + anti-CD28 (1 µg/mL) antibodies for 6 hours. Expression of AR, IFNγ and IL-4 in CD4 T cells was analyzed by ICS. Biotinylated goat anti-mouse AR antibodies were obtained from R&D Systems. LEAF™ purified anti-mouse CD3ε (145-2C11) and LEAF™ purified anti-mouse CD28 (37.51) were purchased from BioLegend. APC-Cy7 conjugated anti-mouse CD3 (17A2), Alexa Fluor 700 conjugated anti-mouse CD4 (GK1.5), Pacific Blue conjugated anti-mouse CD44 (IM7), PerCP-Cy5.5 conjugated anti-mouse CD69 (H1.2F3), APC conjugated anti-mouse IL-2 (JES6-5H4), PE-Cy7 conjugated anti-mouse IL-4 (BVD6-24G2), PE conjugated anti-mouse IL-5 (TRFK5), and FITC-conjugated streptavidin were obtained from eBioscience. PE-Alexa Fluor 610 conjugated anti-mouse IFNγ (XMG1.2) was obtained from Invitrogen. Similar results were obtained in at least three experiments.
(TIF)
