# Supplementary figures and images for: The Acute Environment, Rather than T Cell Subset Pre-Commitment, Regulates Expression of the Human T Cell Cytokine Amphiregulin
Source: PLoS One. 2012 Jun 14;7(6):e39072. doi: 10.1371/journal.pone.0039072 (PMC3375254; doi:10.1371/journal.pone.0039072)

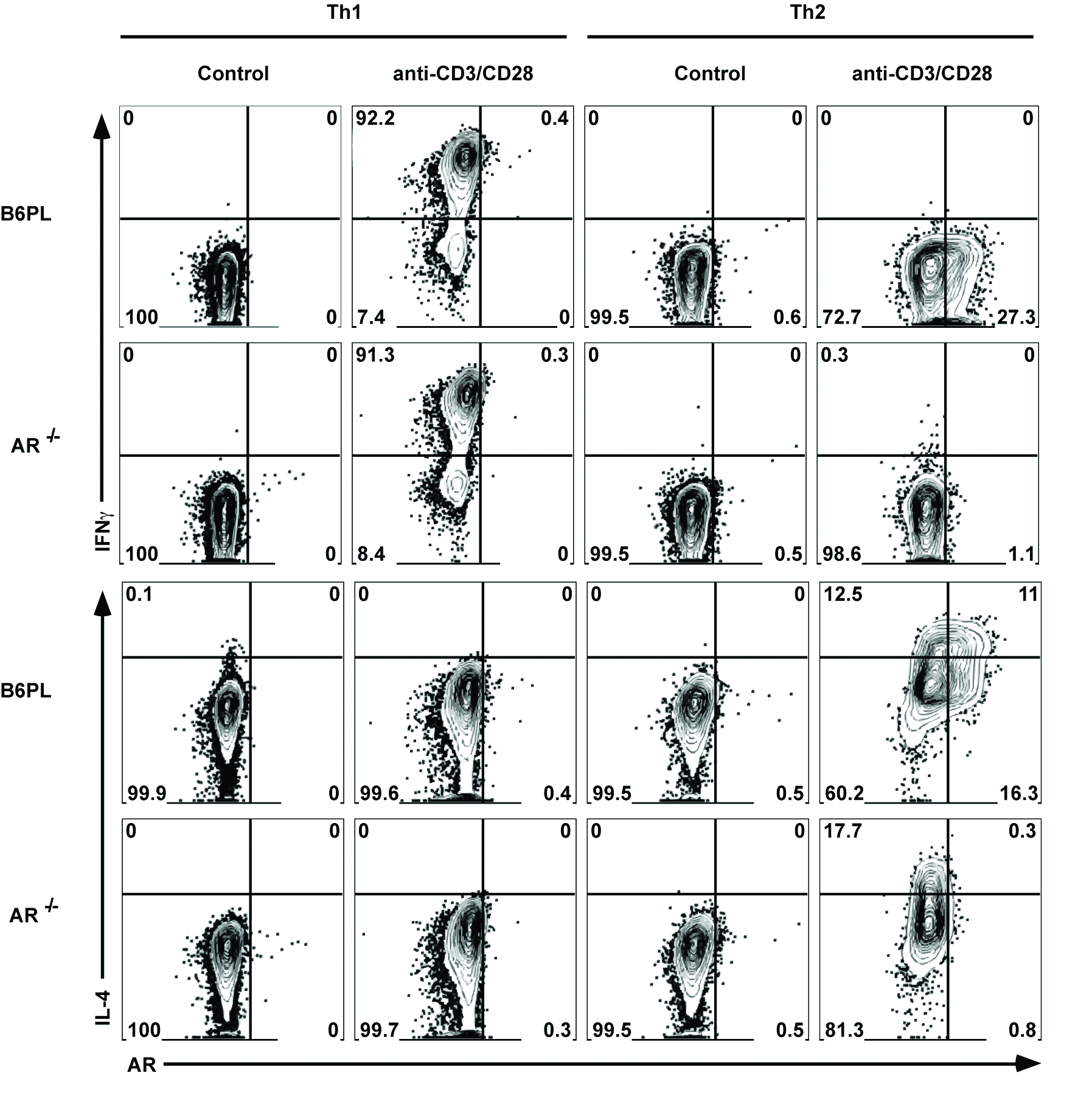

Supplement: Figure S1 — Mouse Th2 but not Th1 cells express AR in response to TCR activation. In vitro induced allogeneic Th1 and Th2 cell lines [50] from B6PL or AR−/− mice were stimulated with plate-coated anti-CD3 (2 µg/mL) + anti-CD28 (1 µg/mL) antibodies for 6 hours. Expression of AR, IFNγ and IL-4 in CD4 T cells was analyzed by ICS. Biotinylated goat anti-mouse AR antibodies were obtained from R&D Systems. LEAF™ purified anti-mouse CD3ε (145-2C11) and LEAF™ purified anti-mouse CD28 (37.51) were purchased from BioLegend. APC-Cy7 conjugated anti-mouse CD3 (17A2), Alexa Fluor 700 conjugated anti-mouse CD4 (GK1.5), Pacific Blue conjugated anti-mouse CD44 (IM7), PerCP-Cy5.5 conjugated anti-mouse CD69 (H1.2F3), APC conjugated anti-mouse IL-2 (JES6-5H4), PE-Cy7 conjugated anti-mouse IL-4 (BVD6-24G2), PE conjugated anti-mouse IL-5 (TRFK5), and FITC-conjugated streptavidin were obtained from eBioscience. PE-Alexa Fluor 610 conjugated anti-mouse IFNγ (XMG1.2) was obtained from Invitrogen. Similar results were obtained in at least three experiments. (TIF) [file pone.0039072.s001.tif]
